# Supplementary material for: Hybrid Models and Biological Model Reduction with PyDSTool
Source: PLoS Comput Biol. 2012 Aug 9;8(8):e1002628. doi: 10.1371/journal.pcbi.1002628 (PMC3415397; doi:10.1371/journal.pcbi.1002628)
Supplement: Text S4 — Complete source code for the PyDSTool package (version 0.88.120504). Includes API documentation and help files linking to web pages. This file is identical to the current public release on Sourceforge.net. (ZIP) [file pcbi.1002628.s004.zip › PyDSTool/html/PyDSTool.Toolbox.optimizers.step.restart_conjugate_gradient-pysrc.html]

xml version="1.0" encoding="ascii"?


PyDSTool.Toolbox.optimizers.step.restart\_conjugate\_gradient


| Home | Trees | Indices | Help | PyDSTool |
| --- | --- | --- | --- | --- |

Package PyDSTool ::
Package Toolbox ::
Package optimizers ::
Package step ::
Module restart\_conjugate\_gradient

[hide private]

[frames] | no frames]

# Source Code for Module PyDSTool.Toolbox.optimizers.step.restart\_conjugate\_gradient

```
 1  
 
 2  # Matthieu Brucher
 
 3  # Last Change : 2007-08-29 11:48
 
 4  
 
 5  """
 
 6  Restarts a conjugate gradient search by deleting the step key in the state dictionary
 
 7  """ 
 8  
 
 9  import random 
10  import numpy 
11  import numpy.linalg 
12  
 


13 -class RestartPeriodicallyConjugateGradientStep(object):


14    """
 
15    A step decorator that periodically deletes the direction key in the dictionary so that the CG search can be restarted
 
16    """ 


17 -  def __init__(self, step, iteration_period = 10):


18      """
 
19      Constructs the decorator
 
20        - step is the step that will be decorated
 
21        - iteration_period is the number of iteration between the deletion
 
22      """ 
23      self.step = step 
24      self.iteration_period = iteration_period

25  
 


26 -  def __call__(self, function, point, state):


27      """
 
28      Computes a step based on a function and a point
 
29      """ 
30      if (state['iteration'] % self.iteration_period == 0) & ('direction' in state): 
31        del state['direction'] 
32      step = self.step(function, point, state) 
33      return step

34  
 


35 -class RestartNotOrthogonalConjugateGradientStep(object):


36    """
 
37    A step decorator that deletes the direction key in the dictionary if the last gradients are not orthogonal enough so that the CG search can be restarted
 
38    """ 


39 -  def __init__(self, step, orthogonal_coeff = 0.1):


40      """
 
41      Constructs the decorator
 
42        - step is the step that will be decorated
 
43        - orthogonal_coeff is the orthogonal limit
 
44      """ 
45      self.step = step 
46      self.orthogonal_coeff = orthogonal_coeff

47  
 


48 -  def __call__(self, function, point, state):


49      """
 
50      Computes a step based on a function and a point
 
51      """ 
52      newGradient = function.gradient(point) 
53      if 'gradient' in state: 
54        if abs(numpy.dot(state['gradient'], newGradient) / numpy.linalg.norm(newGradient)) > self.orthogonal_coeff: 
55          del state['direction'] 
56      step = self.step(function, point, state) 
57      return step

58
```

  


| Home | Trees | Indices | Help | PyDSTool |
| --- | --- | --- | --- | --- |

|  |  |
| --- | --- |
| Generated by Epydoc 3.0.1 on Fri May 4 15:24:15 2012 | http://epydoc.sourceforge.net |
